# Analysis of Few-Shot Techniques for Fungal Plant Disease Classification and Evaluation of Clustering Capabilities Over Real Datasets

**DOI:** 10.3389/fpls.2022.813237

**Published:** 2022-03-07

**Authors:** Itziar Egusquiza, Artzai Picon, Unai Irusta, Arantza Bereciartua-Perez, Till Eggers, Christian Klukas, Elisabete Aramendi, Ramon Navarra-Mestre

**Affiliations:** ^1^TECNALIA, Basque Research and Technology Alliance (BRTA), Parque Tecnológico de Bizkaia, Derio, Spain; ^2^University of the Basque Country, Bilbao, Spain; ^3^BASF SE, Limburgerhof, Germany

**Keywords:** plant disease, convolutional neural network, triplet loss, categorical cross-entropy loss, few-shot learning

## Abstract

Plant fungal diseases are one of the most important causes of crop yield losses. Therefore, plant disease identification algorithms have been seen as a useful tool to detect them at early stages to mitigate their effects. Although deep-learning based algorithms can achieve high detection accuracies, they require large and manually annotated image datasets that is not always accessible, specially for rare and new diseases. This study focuses on the development of a plant disease detection algorithm and strategy requiring few plant images (Few-shot learning algorithm). We extend previous work by using a novel challenging dataset containing more than 100,000 images. This dataset includes images of leaves, panicles and stems of five different crops (barley, corn, rape seed, rice, and wheat) for a total of 17 different diseases, where each disease is shown at different disease stages. In this study, we propose a deep metric learning based method to extract latent space representations from plant diseases with just few images by means of a Siamese network and triplet loss function. This enhances previous methods that require a support dataset containing a high number of annotated images to perform metric learning and few-shot classification. The proposed method was compared over a traditional network that was trained with the cross-entropy loss function. Exhaustive experiments have been performed for validating and measuring the benefits of metric learning techniques over classical methods. Results show that the features extracted by the metric learning based approach present better discriminative and clustering properties. Davis-Bouldin index and Silhouette score values have shown that triplet loss network improves the clustering properties with respect to the categorical-cross entropy loss. Overall, triplet loss approach improves the DB index value by 22.7% and Silhouette score value by 166.7% compared to the categorical cross-entropy loss model. Moreover, the F-score parameter obtained from the Siamese network with the triplet loss performs better than classical approaches when there are few images for training, obtaining a 6% improvement in the F-score mean value. Siamese networks with triplet loss have improved the ability to learn different plant diseases using few images of each class. These networks based on metric learning techniques improve clustering and classification results over traditional categorical cross-entropy loss networks for plant disease identification.

## 1. Introduction

Plants are vulnerable to attack by organisms that interrupt or modify their physiological processes, disrupting plant growth, their development or their vital functions, thus causing plant disease. Plant diseases have a significant impact in agriculture, producing crop yield losses, impairing product quality or limiting availability of food and raw materials. Estimations of global productivity losses are between 20 and 40% annually, and up to 16% of the losses are due to plant diseases (Oerke, 2006). Therefore, plant disease management is essential to reduce crop losses caused by pathogens. Diseases are mainly controlled using chemical fungicides, which in most cases are very efficient (Hirooka and Ishii, 2013). On the other hand, manual plant disease identification is expensive and time-consuming, as it involves human experts to ensure a correct diagnosis. Consequently, automatic plant disease classification algorithms have become a very important and active field of research in agriculture (Sandhu and Kaur, 2019; Shruthi et al., 2019).

Over the years classical computer vision techniques have been widely used for automatic plant disease classification. For instance, Kim et al. (2009) classified grapefruit peel disease using color texture feature analysis under laboratory conditions. Camargo and Smith (2009) developed an image-processing algorithm to identify visual symptoms of plant disease. Revathi and Hemalatha (2012) used edge detection technique to classify cotton leaf diseases. Sannakki et al. (2011) analyzed image color information to predict disease grade on plant leaves. Johannes et al. (2017) developed a early symptom wheat disease diagnosis algorithm for mobile capture devices. Several other algorithms have been developed for different crops, such as rice (Phadikar et al., 2012, 2013), corn (Kiratiratanapruk and Sinthupinyo, 2011), or potato (Dacal-Nieto et al., 2009).

Traditional computer vision approaches depend on the domain knowledge of experts who draft the relevant features for the classification task. This becomes overly complex as the number of crops and diseases increases, compromising the generalizability of the models. This is one of the reasons why Deep Learning (DL) models have replaced classical computer vision techniques in image classification (Picon et al., 2019a) or segmentation (Lin et al., 2019). DL models are frequently based on Convolutional Neural Networks (CNNs). CNNs automatically select most descriptive and salient features for the classification task, and have thousands of adjustable parameters to address complex classification tasks. Thus, CNNs have also been introduced for image based plant disease classification. For example, Sladojevic et al. (2016) created a leaf image classification algorithm to recognize 13 diseases which was also able to differentiate plant leaves from their surroundings. Ferentinos (2018) developed a classification algorithm to distinguish 58 plant species using an open database of more than 85,000 images. Picon et al. (2019a) and Johannes et al. (2017) extended by creating an early disease detection algorithm based on a DL model. Fuentes et al. (2017) presented a DL tomato plant disease and pest detector. There are various recent excellent reviews of DL for plant image classification (Saleem et al., 2019; Hasan et al., 2020; Li et al., 2021).

The importance of plant disease identification algorithms has led to the creation of open access agronomic datasets, for use by agronomists and artificial intelligence researchers as a benchmark to experiment and evaluate new techniques. A salient example is PlantVillage (Hughes and Salathé, 2015), an open access repository of over 50,000 expertly curated images of healthy and infected plant leaves acquired in laboratory conditions. The PlantVillage images include 14 crop types (e.g., cherry, corn, grape, tomato, and pepper) and 26 diseases. Many DL plant disease classification models have been developed and evaluated using the PlantVillage dataset (Mohanty et al., 2016; Rangarajan et al., 2018; Kamal et al., 2019; Too et al., 2019; Argüeso et al., 2020; Mohameth et al., 2020). However, images obtained under laboratory conditions have controlled lighting, smooth backgrounds, and diseases are at an advanced stage of infection. In the field, illumination conditions are uncontrolled, backgrounds are changeable and diseases appear at different stages, including early stages. Early stage disease detection in these challenging conditions is of outmost importance since proper treatment could cure the damage to the crop, but at an early infection stage healthy and diseased plant images are visually very similar. These reasons explain why algorithms developed using the PlantVillage dataset with accuracies over 99% (Mohanty et al., 2016), present accuracies as low as 31.4% when tested on real field images.

In Ghosal et al. (2018), an explainable deep CNN framework was developed to identify, classify and quantify biotic and abiotic stresses in soybean. This framework uses an unsupervised approach to accurately isolate visual symptoms without the need for detailed expert annotation. They identify and classify eight biotic and abiotic soybean stresses by learning from over 25,000 images. Thanks to the application of explainability techniques, they are able to understand the classification decisions made by extracting the visual features learned by the model based on their localized activation levels. These characteristics are then compared with the symptoms identified by humans to validate the results. Another study (Toda and Okura, 2019) also developed a variety of visualization methods using a CNN to understand the network mechanism for disease diagnosis. The attention maps generated by the network found the most significant regions of stressed lesions, matching human decision-making to determine disease. These two studies demonstrated the importance of understanding the mechanism of CNNs for plant stress phenotyping. DeChant et al. (2017) trained several CNNs to classify small regions of maize images as containing northern leaf light (NLB) lesions or not using a sliding window over the images. Predictions from all CNNs were combined into separate heat maps and then fed into a final CNN for stressed lesion detection. The generated heat maps were used as a visualization mechanism to explain classification decisions.

Another limitation of DL models is the need for large annotated datasets to adjust their millions of adjustable parameters. Compiling real field images with disease annotations is very resource consuming, so many efforts are focused on learning from few images, a set of techniques known as Few Shot Learning (FSL). FSL methods for image classification are divided into three main types: data augmentation, transfer-learning, and meta-learning. Data augmentation consists in generating new instances from previous images, for instance using generative adversarial networks (Hu et al., 2019). In transfer learning a baseline network is trained with a large number of images other than the target classes, and then the network is fine-tuned using few instances of the target classes. Typical learning architectures are based on siamese networks and metric learning (distances among classes), as proposed in Argüeso et al. (2020) for plant disease classification. Finally, in meta-learning the models are trained in a set of related prediction tasks, as described by Li and Yang (2021) for plant and pest image classification. Nazki et al. (2020) generated synthetic images to train CNN models for tomato plant disease classification based on generative adversarial networks (GANs). Their model, called AR-GAN, was based on Cycle-GAN (Zhu et al., 2017) and was developed to transform healthy tomato leaves into different types of diseases. They claimed that their technique could improve the performance of plant disease classification compared to other classical data augmentation techniques. AR-GAN was trained on images without complex backgrounds, so this approach might present difficulties in transforming images from datasets taken in the real field.

The objective of this study is to demonstrate a FSL approach based on siamese networks and metric learning trained and evaluated in the challenging conditions of real field images. For that purpose a dataset of real field images containing 5 crops and 17 diseases was used, and experiments were conducted to evaluate how small a dataset could be used to obtain acceptable classification results. Our results show that FSL methods based on siamese networks outperform classical CNN learning methods when trained with less than 200 images per class.

## 2. Materials

The study dataset was compiled in the 2014–2019 period in three phases and at different farmlands in Germany and Spain, as described in Johannes et al. (2017), Picon et al. (2019a), and Picon et al. (2019b). Images were acquired using different electronic devices (e.g., iPhone4, iPhone5, Samsung Galaxy Note, and Windows Phone) throughout the growing season to capture different growth stages of infection.

**Figure 1:**
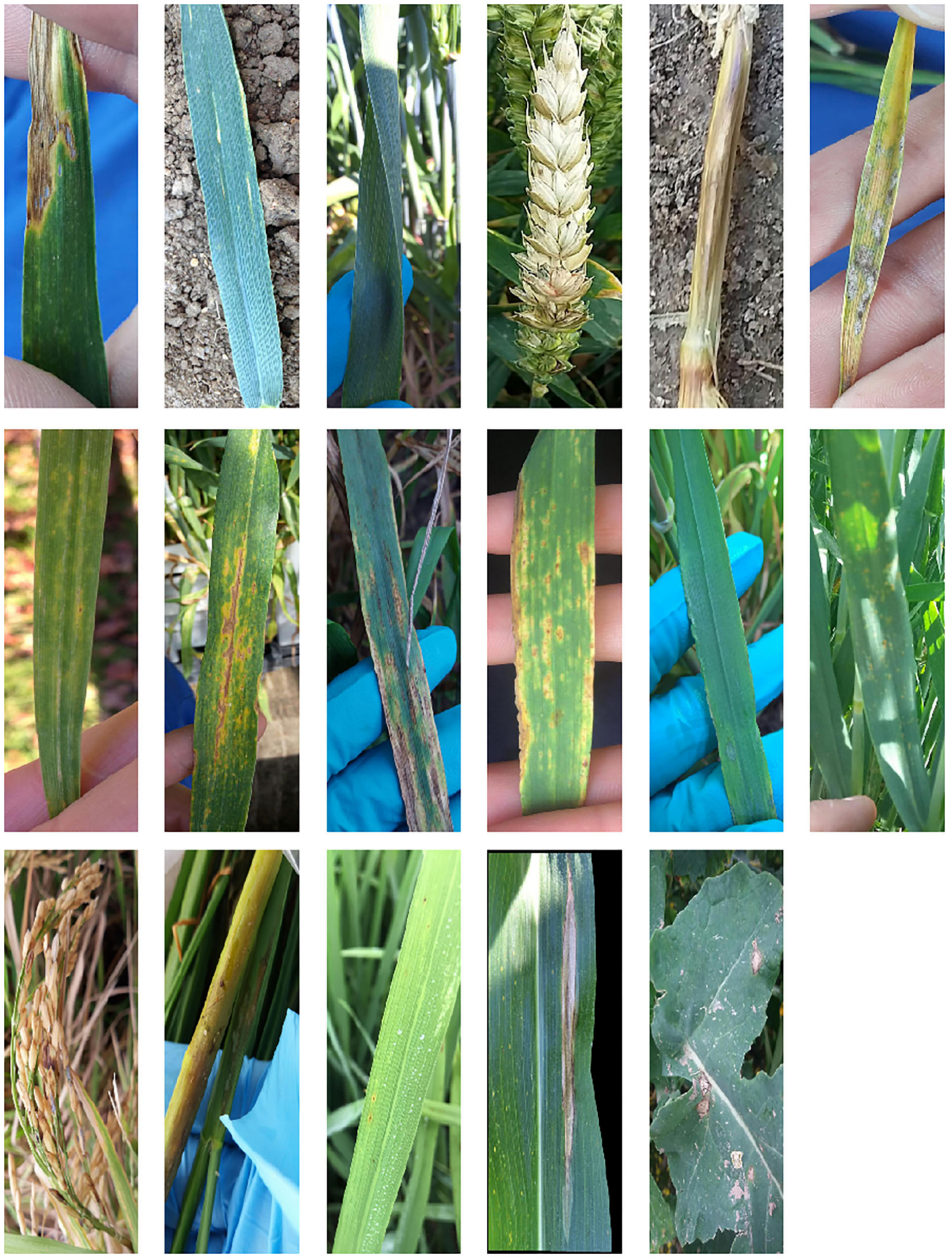
Examples from the 17 diseases in the generated dataset, ordered from left to right by crops: wheat (*Septoria tritici, Puccinia striiformis, Puccinia recondita, Gibberella zeae, Oculimacula yallundae, Blumeria graminis, Septoria nodorum*, and *Drechslera tritici-repentis*), barley (*Pyrenophora teres, Ramularia collo-cygni, Rhynchosporium secalis*, and *Puccinia hordei*), rice (*Various diseases, Thanatephorus cucumeris*, and *Pyricularia oryzae*), corn (*Helminthosporium turcicum*), and rape seed (*Phoma lingam*).

The dataset is composed of 121,955 images of plant leaves, stems and panicles that have been taken by cell phone in real field conditions (Picon et al., 2019b). It contains five types of crops: wheat, barley, rice, corn and rape seed. And in those crops there are 17 representative diseases, including: Rust, Septoria, Tan Spot, Eyespot, Scab, Powdry mildew, Net Blotch, Scald, Blast, Lef blight, or Blackleg. Table 1 provides a detailed composition of the dataset in terms of number of images per crop and disease (causing fungi), and Figure 1 shows an example of each disease.

**Table 1:** Diseases and number of images from the annotated dataset.

**Crop**	**Disease**	**Images**	**Crop**	**Disease**	**Images**
Wheat	Healthy	6,704	Rice	Healthy	4,051
Wheat	Septoria tritici	18,841	Rice	Various diseases	206
Wheat	Puccinia striiformis	15,376	Rice	Thanatephorus cucumeris	2,438
Wheat	Puccinia recondita	16,413	Rice	Pyricularia oryzae	2,441
Wheat	Septoria nodorum	602			
Wheat	Drechslera tritici-repentis	9,550	**Total rice:**		**11,295**
Wheat	Oculimacula yallundae	1,489			
Wheat	Gibberella zeae	1,207	Corn	Healthy	206
Wheat	Blumeria graminis	2,866	Corn	Helminthosporium turcicum	425
**Total wheat:**		**64,026**	**Total corn:**		**631**
Barley	Healthy	1,624	Rape seed	Healthy	6,850
Barley	Pyrenophora teres	15,352	Rape seed	Phoma lingam	6,924
Barley	Ramularia collo-cygni	3,441			
Barley	Rhynchosporium secalis	11,279	**Total rape seed:**		**13,774**
Barley	Puccinia hordei	3,323			
**Total barley:**		**32,229**			
**TOTAL**:					**121,955**

**Figure 2:**
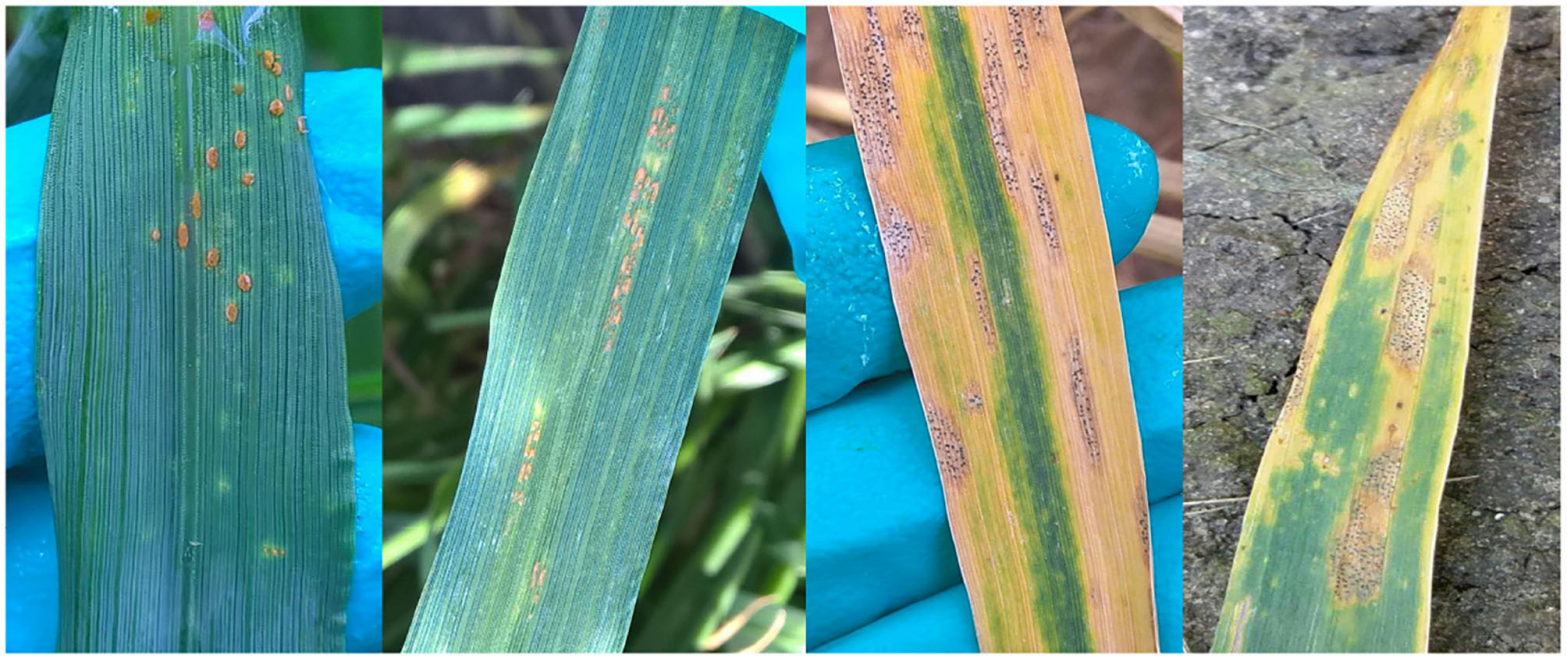
Examples of similar diseases. On the one hand, from left to right: *Puccinia Recondita* and *Puccinia Striiformis*. On the other hand, *Septoria tritici* and *Septoria nodorum*.

The automatic classification of the images in the dataset is complex. Besides the differing illumination and acquisition conditions, the dataset presents several diseases at early stages which are very hard to differentiate, for example *Puccinia recondita* and *Puccinia striiformis* in wheat. Some other diseases present similar symptoms in both early and late stages of infection, like *Septoria tritici* and *Septoria nodorum* in wheat. Figure 2 shows examples illustrating the similarities between those diseases. Moreover, in 9,923 images the crop was infected with various diseases.

This study focuses on single label classification, so images with multiple diseases were discarded. Then the dataset was split into a training (80%), a validation (10%), and a test set (10%). Experiments were conducted with a decreasing number of images per class during training, and once the models were trained the results were obtained for the complete test set. For the experiments, images of different resolution and size have been considered, as different devices have been used to acquire the images.

## 3. Methods

**Figure 3:**
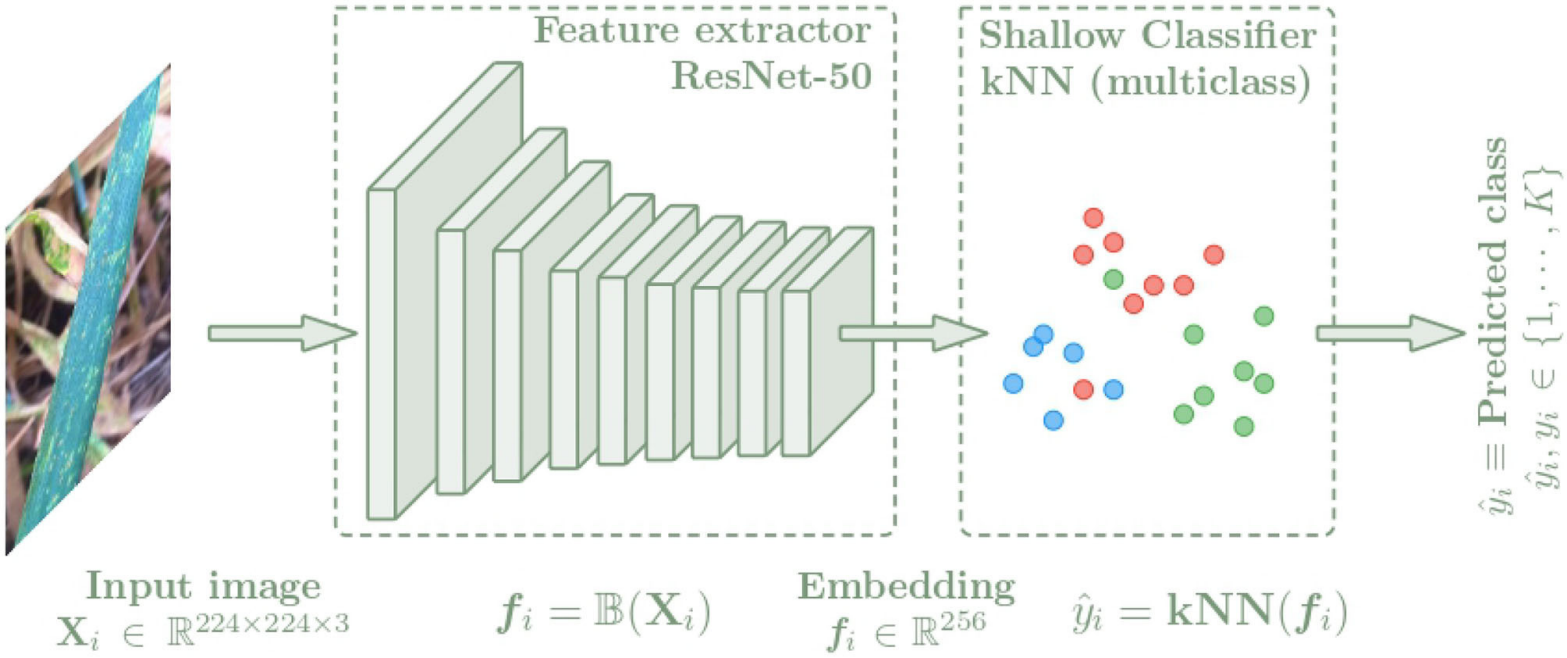
Architecture of the plant disease classification algorithm separated into two blocks. First, a CNN is used to extract features from the input images *X*_*i*_ getting an embedding vector *f*_*i*_ = *f*(*X*_*i*_). Then a *k*-NN classifier is trained with the *f*_*i*_ embeddings to predict the class of each image.

In this section, the architecture used for the plant disease classification algorithm is presented. In order to compare the benefits of metric learning techniques over classical techniques, this architecture is composed of two parts as shown in Figure 3. First, images of leaves, stems or panicles of the specified plant species are used as input to the convolutional neural network (CNN), which is trained to extract features from the images by representing them with an embedding vector. A ResNet-50 (He et al., 2016) neural network has been selected as backbone. To analyze the quality of the generated latent spaces and to obtain the class predictions, a k-nearest neighbors classifier is then used as the shallow classifier, which is fed with the embedding vectors to learn to distinguish the different classes referring to plant disease and providing the final output value of the algorithm. The *k*-nn classifier is a non-parametric method that only depends on the quality of the features and does not interfere with any additional parameters, which has made it a common practice (Wu et al., 2018; Caron et al., 2021).

The idea of this work is to demonstrate that distance metric learning techniques achieve a better vector representation of the images than classical methods when a small dataset is used. We have focused on cases where the supporting dataset, which is often used to train the baseline models before applying the few-shot technique, is not available. Therefore, in this work, models capable of learning classes from a few samples have been developed using a metric learning loss function (triplet loss) and compared with a traditional loss function (cross-entropy loss). Several experiments have been developed using different numbers of training images per class (from *N* = 4 to *N* = 2,000) to evaluate how the network learns with few samples, where two approaches have been compared. On the one hand, a Siamese network with three sub-networks and the Triplet loss function was used to test the metric learning techniques. On the other hand, a traditional single network and the Categorical cross-entropy loss function were used. In both cases the networks worked as feature extractors and then a *k*-NN classifier was added to learn the feature map representations and convert them into class predictions.

### 3.1. Baseline CNN

In our method, the ResNet-50 convolution neural network has been used as the base model and adapted as described in Picon et al. (2019a) to identify diseases from a leaf centered image. This network falls into the subgroup of Residual Networks (*ResNets*) where the main idea is to skip convolutional layer blocks by using shortcut connections. ResNet implements, on the one hand identity blocks that have no convolution layer at shortcut, and do not change dimensions of the feature map, and on the other hand, convolution blocks, which add convolution layer at shortcut, thus increasing the output dimensions with respect to the input. In both cases, batch normalization is performed after each convolution, and then, ReLU activation is applied.

The ResNet-50 consists of 50 layers, with more than 23 million of tuning parameters. It is trained on more than a million images from the ImageNet database (Deng et al., 2009), from which meaningful feature representations have been learnt. In our experiments, the last 33 layers have been unfrozen to adjust the weights to our classification case. After the last layer, a global average pooling operation has been implemented to obtain an image representation of 2,048 features, which has then been reduced to 256 features by adding a neuron layer. Each experiment with the ResNet-50 backbone has been made with two different function losses: triplet loss and categorical cross-entropy loss. Finally, a *k*-NN classifier has been added to the baseline CNN to predict the final class values of the feature embeddings.

### 3.2. Loss Functions

Neural network models use loss functions to calculate the error of the model at each iteration. The algorithm then updates the weights so that the next iteration reduces the previous error, with the aim of minimizing it by means of Stochastic Gradient descent algorithm together with back-propagation. In this sense, the loss function is a fundamental part of a neural network training as it is the mathematical function that guides the training goal for the network.

Image classification neural networks such as ResNet50 are normally used with a cross-entropy loss function which leads to appropriate classification. However, when few samples are available, this loss tends to generate unreliable latent representations (Argüeso et al., 2020). However, metric learning losses aim to learn feature embeddings from images by applying distance metrics to ensure intra-class compactness and inter-class separability. These embeddings keep the most significant features related to the corresponding class of each image, and the loss function tries to increase the distance between samples of different classes while keeping samples of the same class close together.

In this paper, traditional categorical cross-entropy loss and distance metric-based triplet loss have been used and compared.

#### 3.2.1. Categorical Cross-Entropy Loss

**Figure 4:**
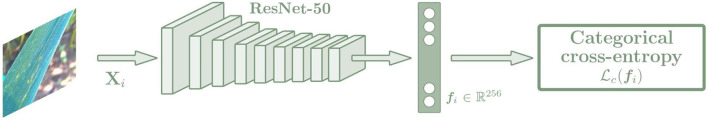
Architecture of the CNN based on the Categorical cross-entropy loss. The network is trained with the input images *X*_*i*_, and the output is an embedding vector *f*_*i*_ of size 256. Then a *k*-NN classifier is trained with the *f*_*i*_ embeddings to predict the class of each image, which is trained in the same way for the triple and categorical models.

Categorical cross-entropy is a common loss function used to solve multi-class classification tasks. The block diagram used with the Categorical cross-entropy loss is shown in Figure 4. This loss function is designed to quantify the difference between two probability distributions. This loss function calculates the loss of an example by applying the following equation:


(1) $$\begin{array}{r} L_{c}=-\underset{i}{\sum }y_{i}\cdot \log\hat{y_{i}}. \end{array}$$

where *y*_*i*_ is the *i*-th real value and $\hat{y_{i}}$ the predicted value by the algorithm. The minus sign ensures that the loss is reduced as the distributions approach each other.

This loss function, together with the architecture defined in Section 3.1 and adapted to identify diseases from a leaf centered image as it was described in Picon et al. (2019a) will serve as baseline model to compare with the metric learning approach described below.

#### 3.2.2. Triplet Loss

**Figure 5:**
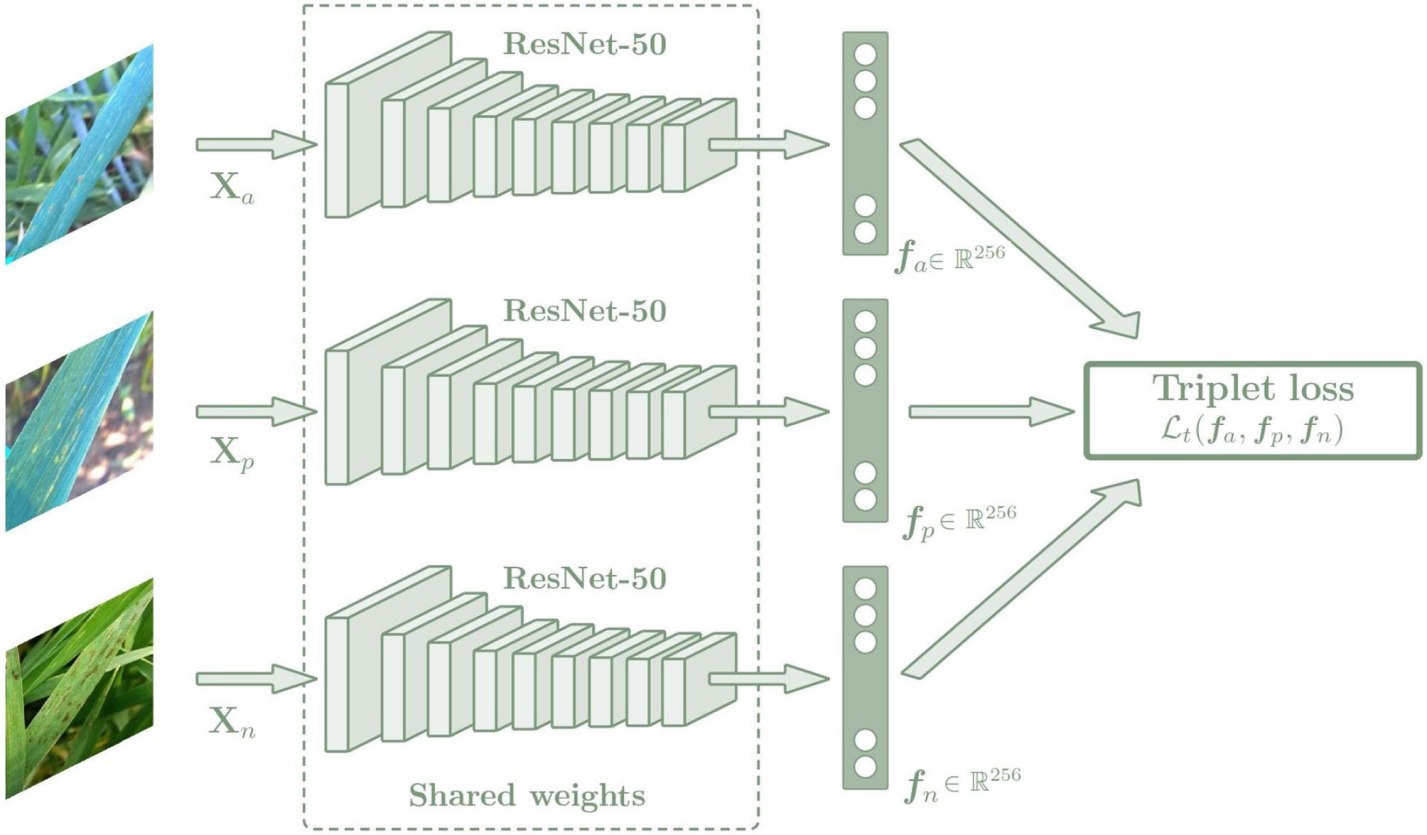
Architecture of the Siamese network based on the Triplet loss. The Siamese network is composed of three sub-networks that share the same weights. The images introduced by these networks must always maintain the same relationship: two of them must belong to the same class, which are the anchor and positive images, and the last one must belong to a different class, which is the negative image. In this way, the Siamese network is trained to minimize the distance between the embeddings of the same class (anchor and positive sample), while maximizing the distance between the embeddings of different classes (anchor and negative sample. The output of the network is an embedding vector *f*_*i*_ of size 256. Then a *k*-NN classifier is trained with the *f*_*i*_ embeddings to predict the class of each image, which is trained in the same way for the triple and categorical models.

Triplet loss function can be used to make the embedding representation more easily separable between classes in a Euclidean vector space. The triplet loss function is used to adjust the network parameters in order to minimize the distance between feature embeddings of the same class, and to maximize the distance between embeddings of different classes at the same time. For this purpose, a Siamese network with three sub-networks is used, where all sub-networks share the same weights and are joined by the triplet loss function (Figure 5). During training, three images of different plants are chosen, which are an anchor (*x*_*a*_), a positive sample (*x*_*p*_), and a negative sample (*x*_*n*_), and each of them is introduced into one of the three sub-networks. In all cases, the anchor and the positive sample belong to the same class while the negative sample belongs to a different class. Image embedding vectors representing the most important features associated with the image class are created as output. The networks compute the distance between the three embedding vectors using the triplet loss function, which is calculated as a Euclidean distance function (Equation 2). Then the parameters of the networks are adjusted to minimize the distance between the embeddings of the anchor and the positive sample, while maximizing the distance between the anchor and the negative sample.


(2) $$\begin{array}{r} L_{t}\left(x_{a},x_{p},x_{n}\right)=\max\left(||f_{a}-f_{p}|{|}^{2}-||f_{a}-f_{n}|{|}^{2}+\alpha ,0\right). \end{array}$$

where ||·||^2^ represents the Euclidean distance and α is a margin between positive and negative pairs, which is used to avoid wasting effort on extending the distance of a negative pair that is distant enough and to focus on more difficult pairs.

### 3.3. *k*-NN Classifier

In order to quantify the classification performance for both approaches, a *k*-nn classifier has been used as a shallow classifier after the feature extraction applied by the neural network. The classifier has been trained using the embedding vectors of the training set for each of the experiments with different neighbor values (*K*). The obtained knn models were used over the embedding vectors from the validation set to select the best *K*-value. This best *K*-value over the validation set was used to quantify the performance of the model in the testing set for class discrimination showing as output one of the 17 plant diseases being analyzed.

In the *k*-nn classification, the output value is selected by a plural vote of its neighbors. Thus, the output is assigned to the most common class among its *k* nearest neighbors, where *k* is a constant value defined by the user, and from which the prediction changes. Therefore, it is important to find the optimal *k*-value. The nearest neighbors can be found using different distance metrics; in this project the Euclidean distance has been applied.

### 3.4. Few-Shot Learning

To demonstrate that neural networks can achieve good results for image classification approaches when a large dataset is not available, several experiments were conducted using different numbers of training images per class, which were randomly selected and ranged from *N* = 4 to *N* = 2, 000. The image features were obtained using a pre-trained network and fine-tuning some layers from the back of the backbone to adjust the weights to the dataset.

In addition, the Triplet and Categorical cross-entropy loss functions were applied separately to create the few shot learning models in order to compare the embedding vectors obtained with the application of each error function. The triplet loss, which is used for distance metric learning, is considered to better learn the embedding representation by keeping objects of the same class close and increasing the distance for objects of different classes.

In all experiments, data augmentation techniques were applied to the training images. Rotations (probability = 0.5, ±90° rotation range), translations (probability = 0.5, ±10%), scaling (probability = 0.7, scale ranges from 50 to 150%) and gamma transformation (probability = 0.5, gamma limits from 80 to 120) were selected. The experiments were conducted over 150 epochs and a learning rate of α = 10^−4^ was selected with the Adam optimizer. The number of training images per class was randomly selected and ranged from *N* = 4 to *N* = 2,000, and all the experiments were run identically for the triplet loss-based model as for the categorical cross-entropy-based model.

### 3.5. Evaluation

The models we have created refer to a multiclass classification problem. Three metrics widely adopted by the scientific community are used for multiclass classification problems such as the recall (*R*_*i*_), precision (*P*_*i*_), and F-score (*F*_1, *i*_) will be employed. These metrics are calculated as follows:


(3) $${\text{R}}_{i}=\frac{N_{ii}}{\sum_{j}N_{ij}},\;{\text{P}}_{i}=\frac{N_{ii}}{\sum_{i}N_{ij}},\;F_{1,\;i}=2\frac{{\text{P}}_{i}\cdot {\text{R}}_{i}}{{\text{P}}_{i}+{\text{R}}_{i}}$$

where *i* refers to the real class (true label), *j* to the predicted class from the algorithm and *N*_*ii*_ and *N*_*ij*_ correspond to the total number of images well-predicted or mixed between them respectively. These predictions are also used to represent the confusion matrix.

Besides that, the results obtained from the feature extractors will also be analyzed. First, a t-distributed Stochastic Neighbor Embedding (t-SNE) method (Van der Maaten and Hinton, 2008) is used to visualize the high-dimensional image features in a two-dimensional graph, which will allow the different clusters to be recognized. On the other hand, Davis-Bouldin index (Davies and Bouldin, 1979) and Silhouette score (Rousseeuw, 1987) clustering metrics are represented. DB index applies quantities and features inherent to the dataset to validate the clustering results, although a good value does not imply the best information retrieval. Lower values of the DB index mean better results. The Silhouette score measures the similarity of an object to its own cluster compared to other clusters. It ranges from −1 to +1, where a high value indicates a better result.

## 4. Results

### 4.1. Training

All experiments were conducted on the training set, consisting of 80% of the full dataset, 10% was used for validation and all results were obtained from the test set, consisting of the other 10%. The distribution was done keeping the percentages of each class and considering the days of taking the images, so that all the images taken on the same day were included in the same set. The ResNet50 neural network pre-trained on the Imagenet dataset was used as the backbone, where the last layers were unfrozen allowing their weights to be modified (as described in Section 3.1). Different experiments were performed using two different loss functions and taking different number of images per class to create few shot learning models. The images were randomly selected and ranged from *N* = 4 images per class to *N*= 2,000. In all experiments, the same number of images was used for each of the disease classes as for the healthy class. In fact, although the collection of images of healthy plants is easier than that of diseased plants, the worst use case was defined and therefore an equal number of healthy images was selected as for the other classes. One experiment was run with each of the selected *N*-values and with each of the two explained loss functions to compare both networks in the few-shot experiments.

For the experiments the images were resized to 224 × 224 pixels, and data augmentation techniques were applied to the training images. Rotations (probability = 0.5), translations, scaling (probability = 0.7) and gamma transformation (probability = 0.5, gamma limits from 80 to 120) were selected. The experiments were conducted over 150 epochs and a learning rate of 10^−4^ was used with the Adam optimizer. The number of training images per class was systematically selected throughout the experiments and ranged from *N* = 4 to *N* = 2,000. All the experiments were run identically for the triplet loss-based model as for the categorical cross-entropy-based models. The output of the network was an embedding vector of 256 features. These vectors represent the ability of each loss function to cluster the different classes and hence the feature extraction capability of the network. In addition, a *k*-NN classifier is then applied to the feature extractor to predict the class related to each embedding so that classification results can also be evaluated.

### 4.2. Classification Results

**Figure 6:**
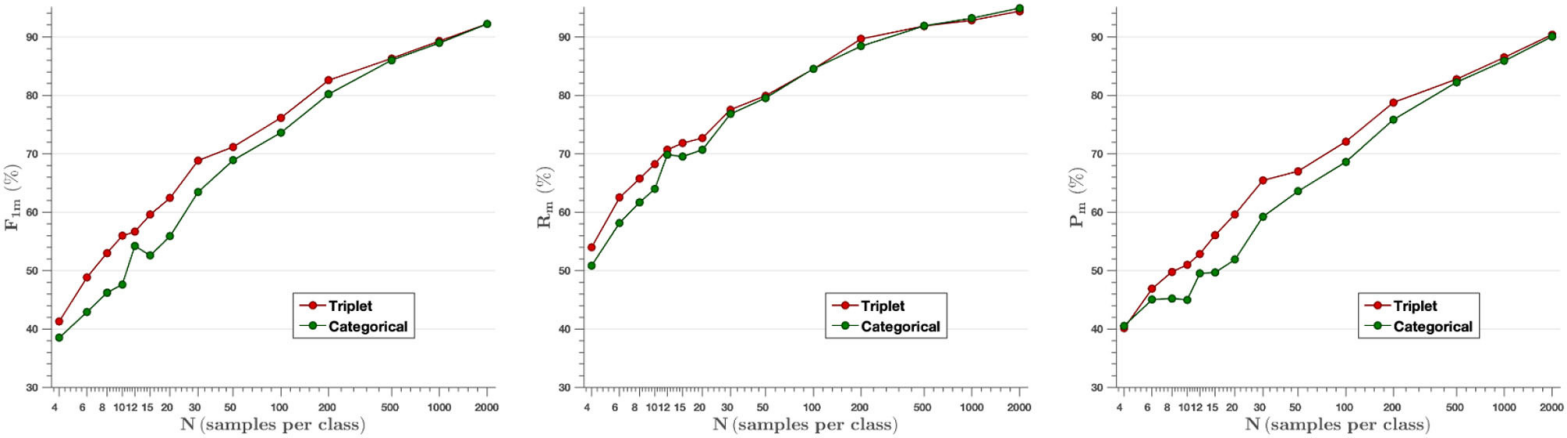
Mean value of F-score (Left), Recall (Middle), and Precision (Right) parameters for all classes as a function of the number of images per class used in training. The results show that the model based on triplet loss outperforms the model based on categorical cross-entropy for experiments from *N* = 4 to *N* = 200 samples per class.

F-score, Recall and Precision parameters have been measured to analyze plant disease classification results. Figure 6 shows the average of the aforementioned metrics for each model created ranging from *N* = 4 images per class to *N*= 2,000. The embedding vectors obtained from the feature extractor have been fed into the *k*-NN classifier, which has been trained and then tested with the testing set. As shown in the first graph, the F-score parameter achieved with the triplet loss outperforms the categorical cross-entropy model up to *N* = 200 samples per class. This difference evens out for higher *N*-values, as the models use more images to train, but at the same time, the difference between them is larger when very few images are used to train (from *N* = 4 to *N* = 30). Thus, comparing the mean F-score values obtained from the F-score results for the different samples per image (*F*1_*triplet*_ = 67.4% vs. *F*1_*cat*_ = 63.6%), we find that the F-score parameter increases by 6% when training with the proposed Siamese architecture and the triplet loss.

**Figure 7:**
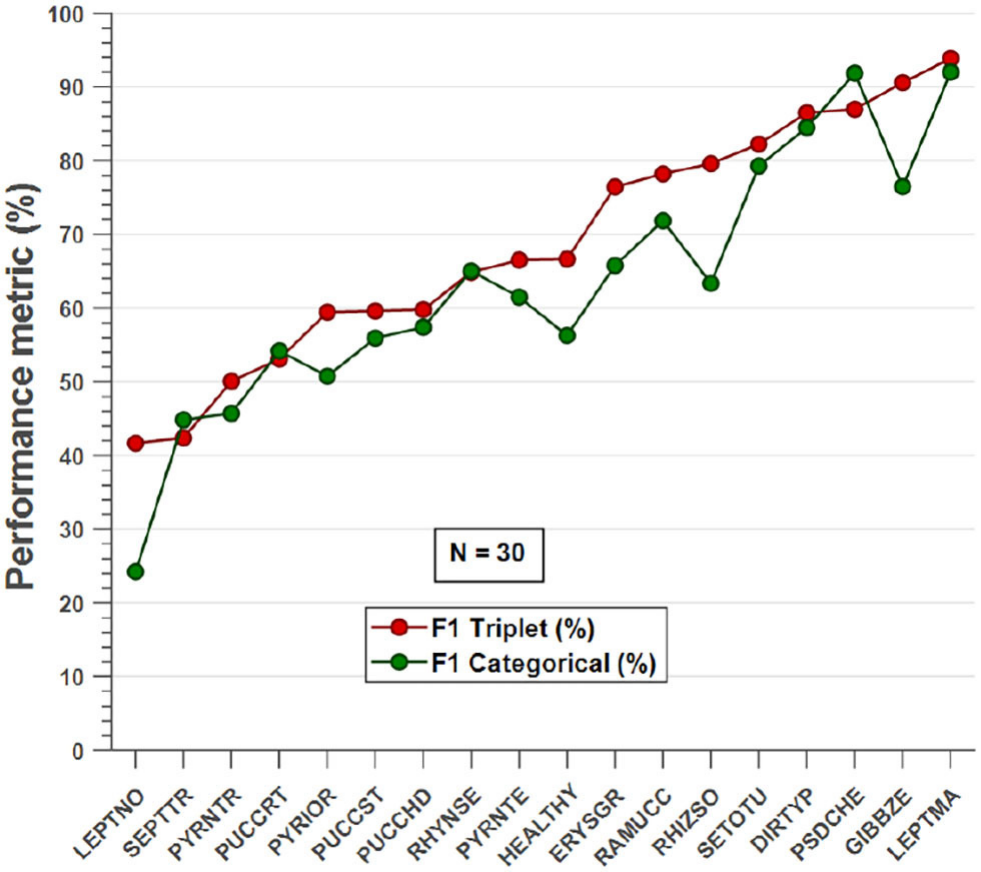
F-score parameter calculated for each class for the case of *N* = 30 images per class. This parameter shows a higher performance for almost all classes when using the triplet loss function.

**Figure 8:**
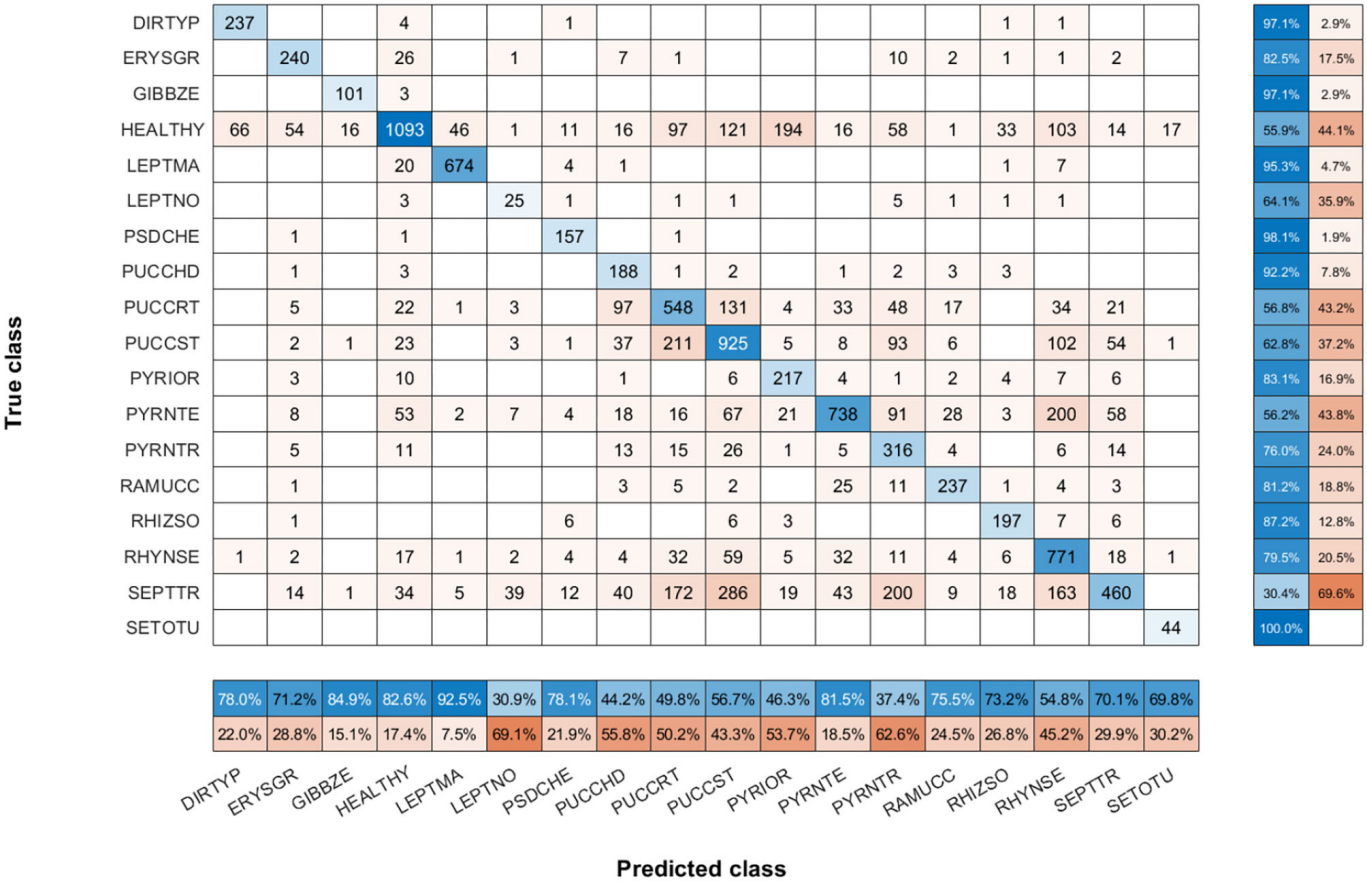
Confusion matrix of *N* = 30 images per class model with triplet loss. The confusion matrix provides an accurate view of how correctly the model predicts the classes or how the classes are misclassified. The values of the diagonal represented in blue correspond to the number of correctly predicted images for each class. The values of the matrix outside the diagonal represented in orange correspond to incorrect predictions, where each cell relates the true class to the class predicted by the algorithm. In addition, below the confusion matrix, the precision values of each class are plotted horizontally in blue. Also, to the right of the confusion matrix, the recall values of each class are shown vertically in blue.

To show the most problematic classes, the F-score parameter has been calculated for each class using the model of *N* = 30 images per class. Figure 7 shows that the F-score parameter is higher for almost all classes by using the triplet loss function. On the other hand, we can analyze that *Septoria nodorum* (LEPTNO) and *Septoria tritici* (SEPTTR) are the classes with the lowest value, below 50% for both Triplet and Categorical cross-entropy losses. For the case of Triplet loss, the confusion matrix has also been calculated to find the predictions of the algorithm for each class (Figure 8). As in the previous case, it can be observed that the class with the worst prediction is *Parastagonospora nodorum* (LEPTNO), which is confused with *Zymoseptoria tritici* (SEPTTR). However, there is no other solution for that as there are not enough images available for LEPTNO and as mentioned above they are two very conflicting classes. From the point of view of plant physiology, both diseases are characterized by the presence of yellowish spots, which quickly turn into gray-brown lesions surrounded by a yellowish once the damage turns brown. In advances stages, the spots may contain small black dots (known as black pycnidia), which are the most characteristic sign of advanced septoria diseases, as shown in Figure 2. One difference is that LEPTNO the pycnidia are smaller, even very difficult to see without the aid of a magnifying glass, and in SEPTTR the pycnidia are visible to the naked eye. SEPTTR is also confused with some other classes, such as *Drechslera tritici-repentis* (PYRNTR), *Puccinia striiformis* (PUCCST), or Puccinia recondite (PUCCRT), which implies that all of them have poor outcomes. In addition, the latter two are also very confusing to each other, since as mentioned above, the different symptoms between them are very subtle. In the early stages of these diseases, individual yellow to orange-brown pustules appear on the leaves. In PUCCRT the pustules tend to be randomly scattered whereas in PUCCST they form in small pockets at the beginning or when the leaves are young, and as the disease progresses, they form in bands. The color of the pustules is usually orange-brown in PUCCRT and orange-yellow in PUCCST.

**Figure 9:**
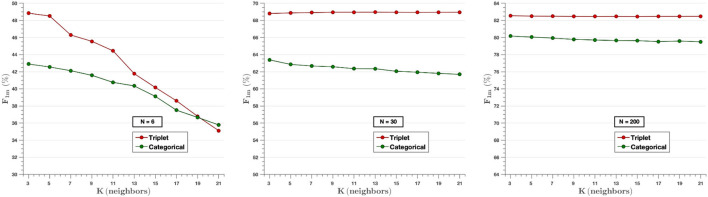
The effect of the *K*-value of the classifier knn for the experiments of *N* = 6 (Left), *N* = 30 (Middle), and *N* = 200 (Right) images per class. In the cases of *N* = 30 and *N* = 200, very little variability is observed for all *K*-values selected. In contrast, in the case of *N* = 6, the value of the F-score decreases as higher *K*-values are chosen.

Figure 9 analyzes the effect of the *K*-value in the *k*-NN classifier for three different experiments (*N* = 6, *N* = 30, *N* = 200), where the parameter F-score is calculated for different values of *K* (3, 5, …, 21). It can be appreciated that Triplet approach surpasses the performance of categorical cross-entropy method. The only exception is observed on *N* = 6 where the choice of value of k higher than the number of image per class (*N* = 6) reduces the performance of the experiment, since in that case the classifier takes into consideration samples of different classes for each prediction. On the other hand, when the value of the classifier is lower than the number of images per class, there is little variability in the results, so the value of K does not influence them. That is, when designing the algorithm, it is not possible to set a value of K higher than the number of classes, since, as can be seen in the graph on the left, the F-score decreases in both models as the value of K increases with respect to N. However, for values of N higher than K, it is observed that the use of the triplet loss model achieves better performance.

### 4.3. Clustering Results

**Figure 10:**
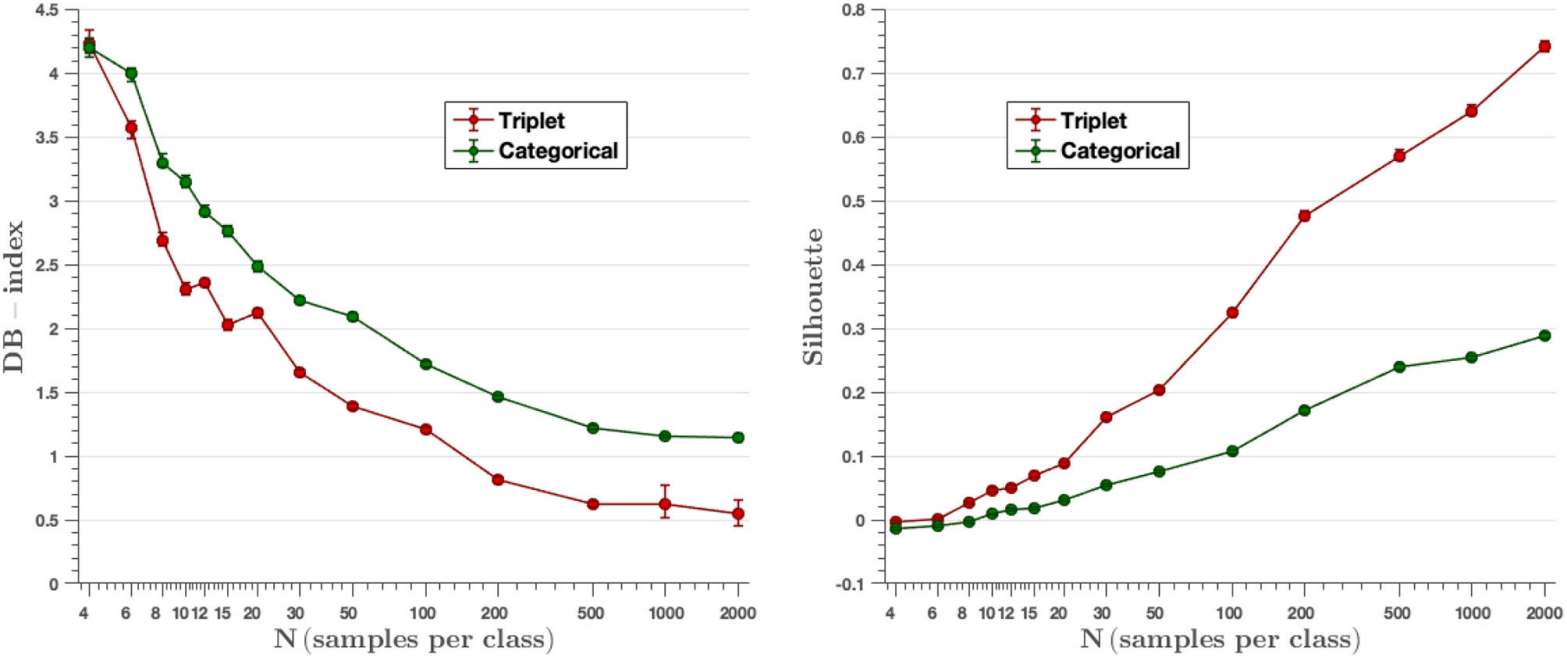
DB-index and Silhouette score metrics. The triplet approach achieves a lower DB-index and a higher Silhouette value than the categorical cross-entropy method for all experiments, resulting in a better ability to group classes into different clusters.

Clusters are generated by the output embedding vectors of the feature extractor. Davis-Bouldin and Silhouette metrics represent the capacity the network has to group the classes in different clusters. Figure 10 displays the values of mentioned parameters for all the models created, where we can see that results improve while the number of images per class increment. Moreover, in all cases, triplet loss models achieve better results for both parameters. The average values of the DB-index and Silhouette parameters have been calculated considering the values obtained in all experiments (from *N* = 4 to *N* = 2,000). On the other hand, we observe that the triplet loss model improves the value by 22.7% with respect to the categorical cross-entropy loss model (*DBindex*_*triplet*_ = 1.87 vs. *DBindex*_*cat*_ = 2.42). Similarly, the Silhouette value also improves, now by 166.7% (*Silhouette*_*triplet*_ = 0.24 vs. *Silhouette*_*cat*_ = 0.09). These metrics are used to assess the quality of the clusters generated by the embedding vectors, and these results show that the triplet loss model achieves better cluster separability than the categorical-cross entropy loss model.

**Figure 11:**
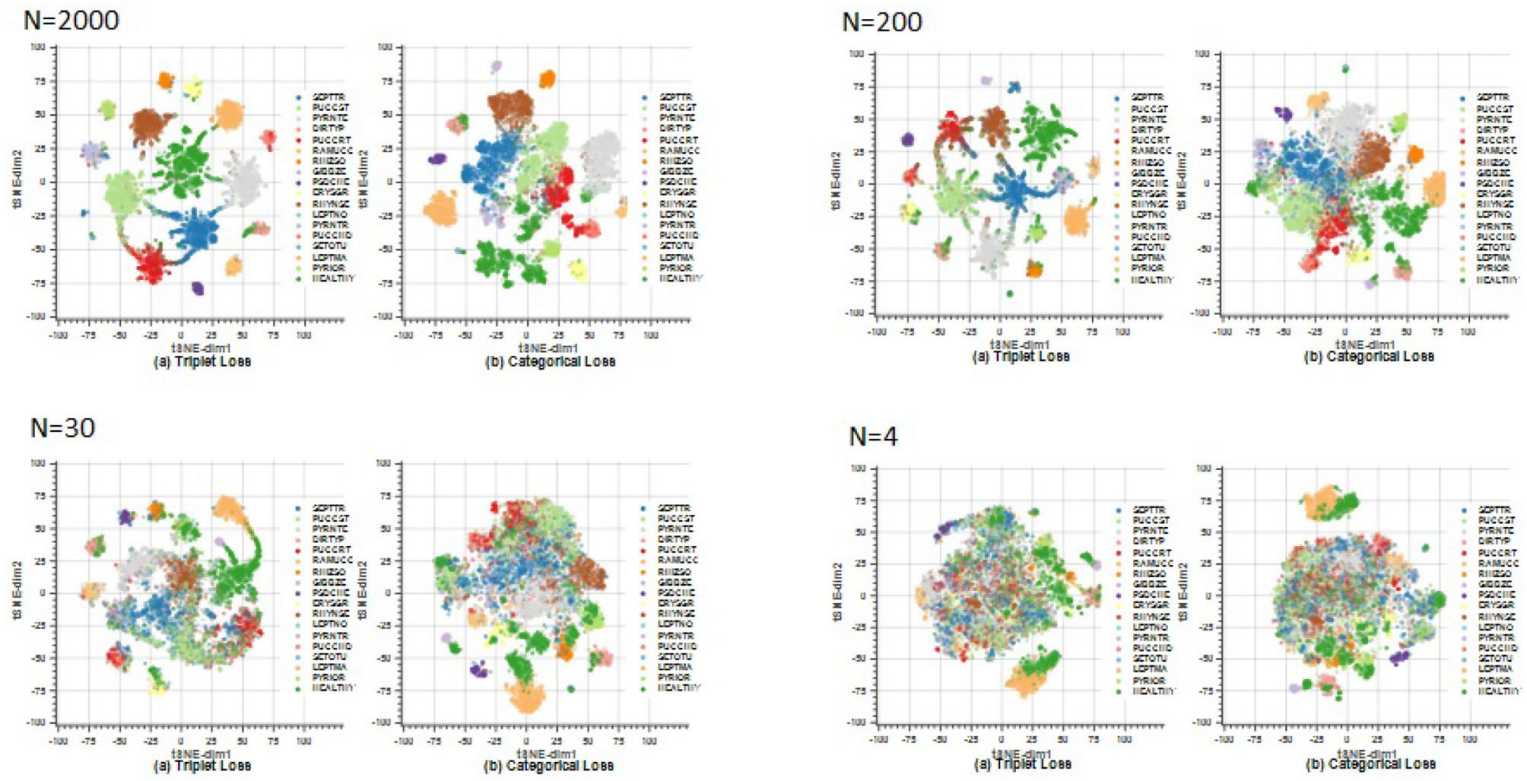
Test embeddings of *N* = 2,000 (top left), *N* = 200 (top right), *N* = 30 (bottom left), *N* = 4 (bottom right) reduced to 2 dimensions using the t-SNE technique. For each experiment, the graphs on the left show the results obtained with the triplet loss, and those on the right show the results obtained with the categorical cross-entropy loss. In the case of *N* = 2,000 and *N* = 200, high class separability is observed with triplet loss. By reducing the number of images per class to *N* = 30, the triplet loss model loses separability in certain classes. Finally, in the case of *N* = 4, the class groups are not well-defined. In the case of categorical cross-entropy loss, similar results are shown in all graphs.

The t-SNE technique obtains the representation of the embedding vectors in a two-dimensional graph. The 256-dimensional embedding vectors are then reduced to two dimensions in order to visualize the clustering capabilities of the different losses to group the test embeddings into class clusters. Figure 11 presents the t-SNE graph of the models created during training, showing the results obtained with the triplet loss models in the left column and the results with the categorical cross-entropy loss models in the right column. Four experiments have been plotted: on the top left, the model results using 2,000 images per class; on the top right, the model results using 200 images per class; on the bottom left, the model results using 30 images per class; and on the bottom right, the model results using 4 images per class. Each color represents a different class. We can see that in the case of *N* = 2,000, the model trained with the triplet loss maximizes the interclass distance achieving a high separability between classes, while minimizing the intraclass distance, creating a grouped cluster of each of the classes. It can also be observed how the SEPTTR class represented in dark blue is close at certain points to several clusters belonging to the PUCCST (light green) or PUCCRT (red) classes, which confuses the algorithm, as analyzed in the results of section 4.5. On the other hand, the t-SNE 2D projection of the model trained with categorical cross-entropy shows a larger overlap between classes which is consistent with the DB index and Silhouette values. By reducing the number of images to 200, a clear difference between the two models is observed. In the case of triplet loss, similar results are obtained with respect to the previous model analyzed (*N* = 2,000). In fact, the model already obtains compact clusters with *N* = 200 and improves slightly when training with more images. In the case of *N* = 30, as before, we obtain a better separability between classes by training the model with triplet loss, since in the case of categorical cross-entropy loss very few classes are well-defined. However, if we compare this with the previous cases of *N* = 200 and *N* = 2,000, we observe that for both models, the experiments trained with 200 and 2,000 images per class achieve a better clustering of the classes with respect to the experiments trained with 30 images per class. This is due to the fact that, by training with a larger number of images the models manage to extract the most representative features of each class which are reflected in the embedding vectors. Finally, in the case of *N* = 4, similar results are obtained with both triplet and categorical cross-entropy loss, where no class separability is shown in either case.

### 4.4. Statistical Analysis

To calculate the statistical significance of the performance of the two proposed algorithms, we follow the approach proposed by Dietterich (1998), where the use of the McNemar test is recommended in cases where multiple iterations of the test are not possible or are time-consuming. McNemar's test proposes a tabulation of the responses given by two proposed qualifiers (in our case the triplet loss and categorical cross-entropy algorithms) where their discrepancies are measured for marginal homogeneity.

Since McNemar's test is aimed at binary decision classifiers, we employ the Stuart-Maxwell test (Maxwell, 1970) which is an extension of McNemar's test for multiclass classification algorithms (Cano-Espinosa et al., 2020). Table 2 details the results obtained. We obtain statistical significance (*p*_*value*_ < 0.01) for all experiments involving less than 500 images for training, while there is no statistical significance for experiments with a number of images greater than 500. This demonstrates the benefit of using metric learning approaches for few-shot learning compared to classical metrics, as also seen in other fields (Medela and Picon, 2019; Argüeso et al., 2020).

**Table 2:** This table shows the statistical significance of the differences among the two proposed classifiers by a different number of training images.

**No of images**	**4**	**6**	**8**	**10**	**12**	**15**	**20**	**30**	**50**	**100**
*p*_*value*_	0.00	0.00	0.00	0.00	0.00	0.00	0.00	0.00	0.00	0.00
χ^2^	0963.26	1888.42	2101.88	2513.66	1044.49	1590.29	1552.43	1056.37	906.48	959.85
**No of images**	**200**	**500**	**1k**	**2k**						
*p*_*value*_	0.00	0.00	0.38	1.00						
χ^2^	280.09	209.58	157.85	102.98						

*It can be appreciated that the differences are significant (p_value_ < 0.01) for training image numbers lower than 500 whereas classifier differences are not significant with larger number of training images*.

### 4.5. Analysis of Explainability

The dataset used to develop the project contains images of plants taken in the field. Therefore, images containing human digits with or without blue gloves are included, which could affect the results of the experiments. The images were captured in different campaigns and at different locations, and in all cases the same protocols were followed for the acquisition of images of all crops so that the occurrence of artifacts was controlled. Additionally, along with the acquisition campaigns, the pictures taken on the same day were assigned to a unique dataset subset (train, validation, or test) to avoid data contamination.

**Figure 12:**
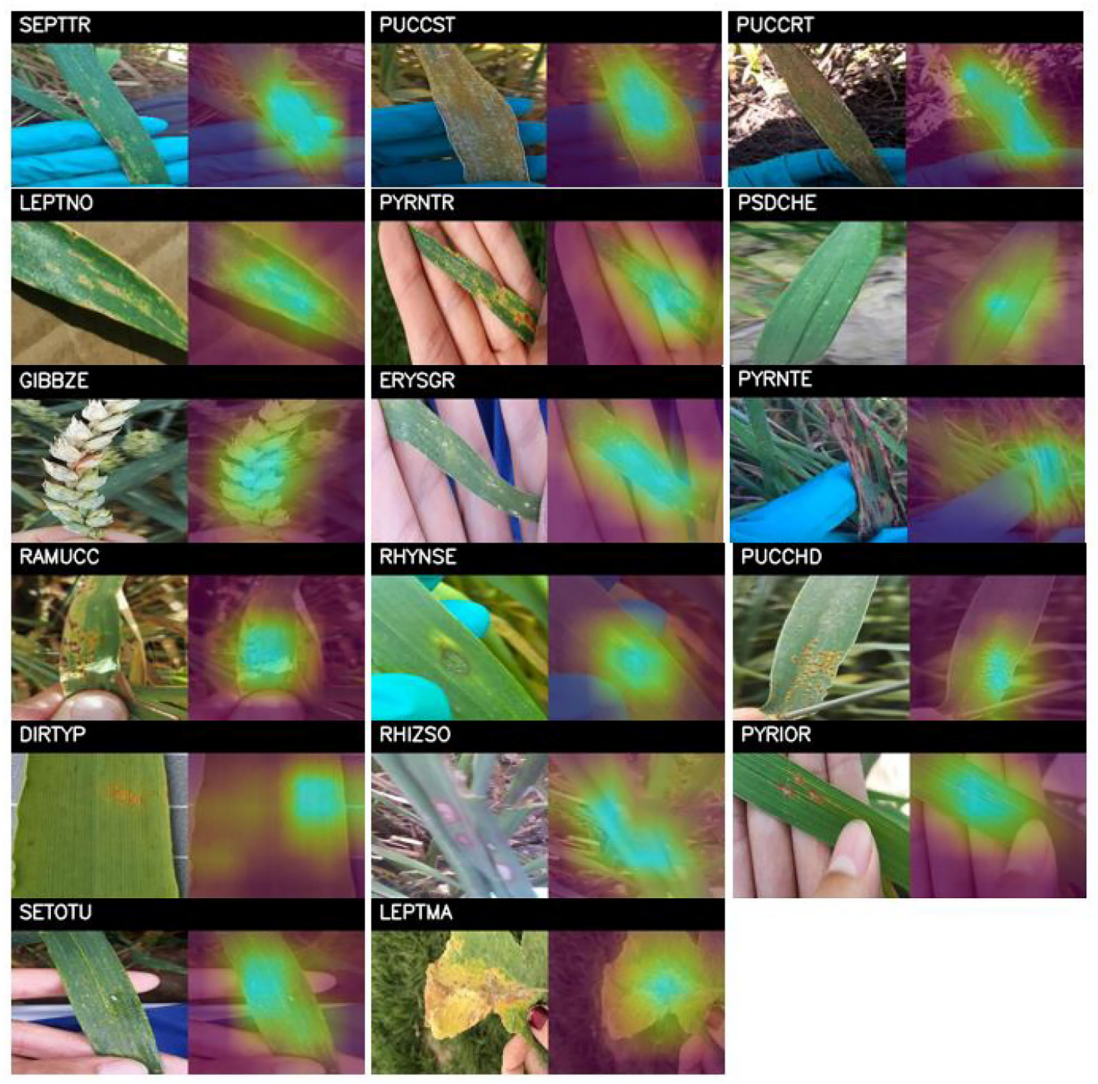
Grad-CAM results applied to test images of all diseases. For each disease, the original image has been plotted on the left, and the most significant regions detected by the algorithm is represented on the right. Each disease is expressed by its EPPO code: SEPTTR (*Septoria tritici*), PUCCST (*Puccinia striiformis*), PUCCRT (*Puccinia recondita*), LEPTNO (*Septoria nodorum*), PYRNTR (Drechslera tritici-repentis), PSDCHE (*Oculimacula yallundae*), GIBBZE (*Gibberella zeae*), EYRSGR (*Blumeria graminis*), PRYNTE (*Pyrenophora teres*), RAMUCC (*Ramularia collo-cygni*), RHYNSE (*Rhynchosporium secalis*), PUCCHD (*Puccinia hordei*), DIRTYP (*Various diseases*), RHIZSO (*Thanatephorus cucumeris*), PYRIOR (*Pyricularia oryzae*), SETOTU (*Helminthosporium turcicum*), and LEPTMA (*Phoma lingam*).

To analyze the influence of these artifacts on the algorithm performance, the Grad-CAM technique (Selvaraju et al., 2017) has been selected, which produces visual explanations for the CNN-based model decisions. It uses the gradients leading to the final convolutional layer to produce a coarse localization map that highlights important regions of the image for prediction. The Grad-CAM technique has been applied to the trained models and test set images to find the most significant regions of the images that the model focused on to predict plant diseases. Figure 12 shows the results obtained on different images for all the diseases, where it can be seen that the trained model correctly focuses on the most representative parts of the diseases, without being affected by the different artifacts such as gloves, human hands or specific backgrounds that might appear on the image.

## 5. Discussion

In recent years, deep learning-based models for plant disease detection have become increasingly important. Thus, some datasets have been created and made publicly available for research. An example of an open access dataset is the PlantVillage dataset, which consists of over fifty thousand images of 26 different diseases that have been taken under controlled conditions. Images of individual leaves are photographed on a plain background where diseases are clearly visible in most cases, as only late stage diseases have been considered. Several experiments have been developed using the PlantVillage dataset and have achieved high performance (Mohanty et al., 2016; Rangarajan et al., 2018). In addition, few-shot learning models have also been applied to this dataset in order to address the problems of acquiring large datasets. Argüeso et al. (2020) reached a median accuracy of 80% for the 6 classes selected in the target dataset using only 15 images per class. However, this model was pre-trained with all class images from the source dataset which acted as a supporting dataset. Therefore, with our experiments we want to demonstrate that by using distance metric techniques good results can be achieved using few images without needing a large dataset of annotated images on which to train the base model. Moreover, the experiments have been developed using images from a dataset taken in real field conditions, which differs from a laboratory dataset by having varied and non-uniform backgrounds, different lighting conditions, different perspectives and distances, as well as by including different disease stages (early and late stages). Five crops with a total of 17 different diseases are included in the dataset.

Our experiments compare two different approaches: a Siamese network based on the distance metric with a triplet loss function, and a traditional network with the categorical cross-entropy loss function. Different models have been developed using from *N* = 4 to *N* = 2,000 images per class. The results show that the Siamese network with the triplet loss function achieves an average f-score above 55.0% from *N* = 10, while the values obtained with the categorical cross-entropy are below in most cases (Figure 6). By increasing the number of classes to *N* = 30, the triplet loss achieves an F-score of 69%, which, considering that there are still very few images, is a great improvement. The effect of the parameter k of the *k*-NN classifier has been analyzed, where it has been observed that the algorithm keeps the results constant for different values of k, except for the cases where K is larger than N for low values of N, in which the effect of the parameter k is large (Figure 9). On the other hand, the results of the feature extractor part of the model have been analyzed. We have analyzed the ability to create clusters of each class by means of the embedding vectors obtained from the CNN through the DB-index and Silhouette parameters, as well as by applying the t-SNE technique. In both cases it has been observed that the Siamese network with the loss of the triplet separates the different classes better, obtaining a clear cluster for each class (Figure 11).

We have performed a statistical analysis to find the most significant differences between the two approaches. In the range from *N* = 4 to *N* = 500, it has been observed that triplet loss-based method outperforms the results obtained with the categorical cross-entropy loss model. For example, in the intermediate value of *N* = 30, we can appreciate that we obtain better results both for classification performance (F1 = 0.69 vs. F1 = 0.63) as well as for clustering performance (DB-Index = 2.25 vs. DB-Index = 1.62) and Silhouette (0.17 vs. 0.05) for the triplet loss approach. For *N*-values above 500, differences are not statistically significant.

## 6. Conclusions

This study analyzes two different networks to develop a model based on deep learning techniques from a few images for plant disease classification: a Siamese network based on the distance metric with a triplet loss function, and a traditional network with the categorical cross-entropy loss function as defined by Picon et al. (2019a).

The experiments have been developed using few images per class. It is noteworthy that we stand for the most complicated case where there is no supporting dataset for creating the few-shot latent descriptor as it is performed in the classical few-shot approaches. For this reason, in this study we have sought to demonstrate that a distance metric-based Siamese network with a triplet loss function is able to learn image features from few images without the need for a supporting dataset which is a more realistic and demanding few-shot use case.

The triplet loss model improves the average F-score value by 6% with respect to the categorical cross-entropy loss. The triplet loss model achieves higher F-score values for all values of N, where the main difference between the two architectures appears at the lowest values of N. Furthermore, it has been analyzed that this difference is due to the fact that the triplet loss model is able to learn the features of classes with fewer available samples and similar symptoms, considered as the most difficult classes. Without loss of generality, in the particular case of *N* = 30, the proposed method outperformed the baseline method for disease classification (F1 = 0.69 vs. F1 = 0.63, *N* = 30). The classes that benefited most from these improvements were LEPTNO and PYRIOR among the most problematic classes, as well as ERYSGR, RHIZSO, or GIBBZE among the classes with the best predictions.

If we analyze the quality of the generated latent space descriptors, we can appreciate that the triplet loss model outperforms the cross-entropy categorical loss model by obtaining more compact and separated clusters (DB-Index = 2.25 vs. DB-Index = 1.62), Silhouette (0.17 vs. 0.05) which allows for easier feature extraction and image retrieval. The triplet loss model improves the mean value of the DB-index parameter by 22.7% over the categorical cross-entropy model, as well as the mean value of the Silhouette score by an improvement of 166.7%.

An important remark when using knn as shallow classifier after the feature extraction that must be taken into account is that the value selected for K must be greater than the number of images per class used for training. In addition, it has also been shown that the results obtained by the classifier are better in the case of triplet loss.

Our results show that triplet loss approach obtains better results than state of the art deep learning approaches for both discriminating plant diseases and generating better latent descriptors in the case of real and complex dataset taken in real field implying complex conditions (changing backgrounds, different lighting conditions, different distances, different disease stages…) where only few images per class are available.

This generates new research opportunities for the use of these techniques in the generation of large and openly available feature extraction models that could help structure plant disease representations allowing few-shot characterization of uncommon and rare diseases.

## Data Availability Statement

The data analyzed in this study is subject to the following licenses/restrictions. The dataset used in this article has been generated by the BASF R&D field research community. It could be made available on reasonable request for non-commercial research purposes and under an agreement with BASF. Requests to access these datasets should be directed to ramon.navarra-mestre@basf.com.

## Author Contributions

IE: conceptualization, investigation, software, formal analysis, methodology, and writing—original draft, review, and editing. AP: conceptualization, investigation, software, methodology, and writing—review and editing. UI: conceptualization, formal analysis, investigation, and writing—review and editing. AB-P: investigation and writing—review and editing. TE and EA: contextualization and writing—review and editing. CK: methodology, software, and writing—review and editing. RN-M: investigation, methodology, and writing—review and editing. All authors contributed to the article and approved the submitted version.

## Funding

This project was partially supported by the Spanish Government through CDTI Centro para el Desarrollo Tecnológico e Industrial project AI4ES (ref CER-20211030), by the University of the Basque Country (UPV/EHU) under grant COLAB20/01 and by the Basque Government through grant IT1229-19.

## Conflict of Interest

IE, AP, and AB-P were employed by TECNALIA. TE, CK, and RN-M were employed by BASF SE. The remaining authors declare that the research was conducted in the absence of any commercial or financial relationships that could be construed as a potential conflict of interest.

## Publisher's Note

All claims expressed in this article are solely those of the authors and do not necessarily represent those of their affiliated organizations, or those of the publisher, the editors and the reviewers. Any product that may be evaluated in this article, or claim that may be made by its manufacturer, is not guaranteed or endorsed by the publisher.

## References

[B1] Argüeso D. Picon A. Irusta U. Medela A. San-Emeterio M. G. Bereciartua A.. (2020). Few-shot learning approach for plant disease classification using images taken in the field. Comput. Electron. Agric. 175, 105542. 10.1016/j.compag.2020.105542

[B2] Camargo A. Smith J. (2009). An image-processing based algorithm to automatically identify plant disease visual symptoms. Biosyst. Eng. 102, 9–21. 10.1016/j.biosystemseng.2008.09.03031297617

[B3] Cano-Espinosa C. González G. Washko G. R. Cazorla M. Estépar R. S. J. (2020). Biomarker localization from deep learning regression networks. IEEE Trans. Med. Imaging 39, 2121–2132. 10.1109/TMI.2020.2965486 31940523 PMC7307703

[B4] Caron M. Touvron H. Misra I. Jégou H. Mairal J. Bojanowski P. Joulin A. (2021). Emerging properties in self-supervised vision transformers. arXiv preprint arXiv:2104.14294.

[B5] Dacal-Nieto A. Vázquez-Fernández E. Formella A. Martin F. Torres-Guijarro S. González-Jorge H. (2009). A genetic algorithm approach for feature selection in potatoes classification by computer vision, in 2009 35th Annual Conference of IEEE Industrial Electronics (Porto: IEEE), 1955–1960.

[B6] Davies D. L. Bouldin D. W. (1979). A cluster separation measure. IEEE Trans. Pattern Anal. Mach. Intell. 1, 224–227. 10.1109/TPAMI.1979.4766909 21868852

[B7] DeChant C. Wiesner-Hanks T. Chen S. Stewart E. L. Yosinski J. Gore M. A.. (2017). Automated identification of northern leaf blight-infected maize plants from field imagery using deep learning. Phytopathology 107, 1426–1432. 10.1094/PHYTO-11-16-0417-R 28653579

[B8] Deng J. Dong W. Socher R. Li L.-J. Li K. Fei-Fei L. (2009). Imagenet: a large-scale hierarchical image database, in 2009 IEEE Conference on Computer Vision and Pattern Recognition (Miami, FL: IEEE), 248–255.

[B9] Dietterich T. G. (1998). Approximate statistical tests for comparing supervised classification learning algorithms. Neural Comput. 10, 1895–1923. 10.1162/089976698300017197 9744903

[B10] Ferentinos K. P. (2018). Deep learning models for plant disease detection and diagnosis. Comput. Electron. Agric. 145, 311–318. 10.1016/j.compag.2018.01.009

[B11] Fuentes A. Yoon S. Kim S. C. Park D. S. (2017). A robust deep-learning-based detector for real-time tomato plant diseases and pests recognition. Sensors 17, 2022. 10.3390/s17092022 28869539 PMC5620500

[B12] Ghosal S. Blystone D. Singh A. K. Ganapathysubramanian B. Singh A. Sarkar S. (2018). An explainable deep machine vision framework for plant stress phenotyping. Proc. Natl. Acad. Sci. U.S.A. 115, 4613–4618. 10.1073/pnas.1716999115 29666265 PMC5939070

[B13] Hasan R. I. Yusuf S. M. Alzubaidi L. (2020). Review of the state of the art of deep learning for plant diseases: a broad analysis and discussion. Plants 9, 1302. 10.3390/plants9101302 33019765 PMC7599890

[B14] He K. Zhang X. Ren S. Sun J. (2016). Deep residual learning for image recognition, in Proceedings of the IEEE Conference on Computer Vision and Pattern Recognition, Las Vegas, NV, 770–778.32166560

[B15] Hirooka T. Ishii H. (2013). Chemical control of plant diseases. J. Gen. Plant Pathol. 79, 390–401. 10.1007/s10327-013-0470-6

[B16] Hu G. Wu H. Zhang Y. Wan M. (2019). A low shot learning method for tea leaf's disease identification. Comput. Electron. Agric. 163, 104852. 10.1016/j.compag.2019.104852

[B17] Hughes D. Salathé M. (2015). An open access repository of images on plant health to enable the development of mobile disease diagnostics. arXiv preprint arXiv:1511.08060.

[B18] Johannes A. Picon A. Alvarez-Gila A. Echazarra J. Rodriguez-Vaamonde S. Navajas A. D.. (2017). Automatic plant disease diagnosis using mobile capture devices, applied on a wheat use case. Comput. Electron. Agric. 138, 200–209. 10.1016/j.compag.2017.04.013

[B19] Kamal K. Yin Z. Wu M. Wu Z. (2019). Depthwise separable convolution architectures for plant disease classification. Comput. Electron. Agric. 165, 104948. 10.1016/j.compag.2019.104948

[B20] Kim D. G. Burks T. F. Qin J. Bulanon D. M. (2009). Classification of grapefruit peel diseases using color texture feature analysis. Int. J. Agric. Biol. Eng. 2, 41–50. 10.3965/j.issn.1934-6344.2009.03.041-050

[B21] Kiratiratanapruk K. Sinthupinyo W. (2011). Color and texture for corn seed classification by machine vision, in 2011 International Symposium on Intelligent Signal Processing and Communications Systems (ISPACS) (Chiang Mai: IEEE), 1–5.

[B22] Li L. Zhang S. Wang B. (2021). Plant disease detection and classification by deep learning–a review. IEEE Access 9, 56683–56698. 10.1109/ACCESS.2021.3069646

[B23] Li Y. Yang J. (2021). Meta-learning baselines and database for few-shot classification in agriculture. Comput. Electron. Agric. 182, 106055. 10.1016/j.compag.2021.106055

[B24] Lin K. Gong L. Huang Y. Liu C. Pan J. (2019). Deep learning-based segmentation and quantification of cucumber powdery mildew using convolutional neural network. Front. Plant Sci. 10, 155. 10.3389/fpls.2019.00155 30891048 PMC6413718

[B25] Maxwell A. E. (1970). Comparing the classification of subjects by two independent judges. Brit. J. Psychiatry 116, 651–655. 10.1192/bjp.116.535.651 5452368

[B26] Medela A. Picon A. (2019). Constellation loss: improving the efficiency of deep metric learning loss functions for optimal embedding. arXiv preprint arXiv:1905.10675. 10.4103/jpi.jpi_41_20 33828896 PMC8020841

[B27] Mohameth F. Bingcai C. Sada K. A. (2020). Plant disease detection with deep learning and feature extraction using plant village. J. Comput. Commun. 8, 10–22. 10.4236/jcc.2020.8600235062534

[B28] Mohanty S. P. Hughes D. P. Salathé M. (2016). Using deep learning for image-based plant disease detection. Front. Plant Sci. 7, 1419. 10.3389/fpls.2016.01419 27713752 PMC5032846

[B29] Nazki H. Yoon S. Fuentes A. Park D. S. (2020). Unsupervised image translation using adversarial networks for improved plant disease recognition. Comput. Electron. Agric. 168, 105117. 10.1016/j.compag.2019.105117

[B30] Oerke E.-C. (2006). Crop losses to pests. J. Agric. Sci. 144, 31–43. 10.1017/S0021859605005708

[B31] Phadikar S. Sil J. Das A. K. (2012). Classification of rice leaf diseases based on morphological changes. Int. J. Inform. Electron. Eng. 2, 460–463. 10.7763/IJIEE.2012.V2.137

[B32] Phadikar S. Sil J. Das A. K. (2013). Rice diseases classification using feature selection and rule generation techniques. Comput. Electron. Agric. 90, 76–85. 10.1016/j.compag.2012.11.001

[B33] Picon A. Alvarez-Gila A. Seitz M. Ortiz-Barredo A. Echazarra J. Johannes A. (2019a). Deep convolutional neural networks for mobile capture device-based crop disease classification in the wild. Comput. Electron. Agric. 161, 280–290. 10.1016/j.compag.2018.04.002

[B34] Picon A. Seitz M. Alvarez-Gila A. Mohnke P. Ortiz-Barredo A. Echazarra J. (2019b). Crop conditional convolutional neural networks for massive multi-crop plant disease classification over cell phone acquired images taken on real field conditions. Comput. Electron. Agric. 167, 105093. 10.1016/j.compag.2019.105093

[B35] Rangarajan A. K. Purushothaman R. Ramesh A. (2018). Tomato crop disease classification using pre-trained deep learning algorithm. Proc. Comput. Sci. 133, 1040–1047. 10.1016/j.procs.2018.07.070

[B36] Revathi P. Hemalatha M. (2012). Classification of cotton leaf spot diseases using image processing edge detection techniques, in 2012 International Conference on Emerging Trends in Science, Engineering and Technology (INCOSET) (Tiruchirappalli: IEEE), 169–173.

[B37] Rousseeuw P. J. (1987). Silhouettes: a graphical aid to the interpretation and validation of cluster analysis. J. Comput. Appl. Math. 20, 53–65. 10.1016/0377-0427(87)90125-7

[B38] Saleem M. H. Potgieter J. Arif K. M. (2019). Plant disease detection and classification by deep learning. Plants 8, 468. 10.3390/plants8110468 31683734 PMC6918394

[B39] Sandhu G. K. Kaur R. (2019). Plant disease detection techniques: a review, in 2019 International Conference on Automation, Computational and Technology Management (ICACTM) (London: IEEE), 34–38.

[B40] Sannakki S. S. Rajpurohit V. S. Nargund V. Kumar A. Yallur P. S. (2011). Leaf disease grading by machine vision and fuzzy logic. Int. J. 2, 1709–1716. 10.1109/SPIN.2015.7095350

[B41] Selvaraju R. R. Cogswell M. Das A. Vedantam R. Parikh D. Batra D. (2017). Grad-CAM: visual explanations from deep networks via gradient-based localization, in Proceedings of the IEEE International Conference on Computer Vision (ICCV), Venice.

[B42] Shruthi U. Nagaveni V. Raghavendra B. (2019). A review on machine learning classification techniques for plant disease detection, in 2019 5th International Conference on Advanced Computing & Communication Systems (ICACCS) (Coimbatore: IEEE), 281–284.

[B43] Sladojevic S. Arsenovic M. Anderla A. Culibrk D. Stefanovic D. (2016). Deep neural networks based recognition of plant diseases by leaf image classification. Comput. Intell. Neurosci. 2016, 3289801. 10.1155/2016/3289801 27418923 PMC4934169

[B44] Toda Y. Okura F. (2019). How convolutional neural networks diagnose plant disease. Plant Phenom. 2019, 9237136. 10.34133/2019/9237136 33313540 PMC7706313

[B45] Too E. C. Yujian L. Njuki S. Yingchun L. (2019). A comparative study of fine-tuning deep learning models for plant disease identification. Comput. Electron. Agric. 161, 272–279. 10.1016/j.compag.2018.03.032

[B46] Van der Maaten L. Hinton G. (2008). Visualizing data using t-SNE. J. Mach. Learn. Res. 9, 2579–2605.

[B47] Wu Z. Xiong Y. Yu S. X. Lin D. (2018). Unsupervised feature learning via non-parametric instance discrimination, in Proceedings of the IEEE Conference on Computer Vision and Pattern Recognition, Salt Lake City, UT, 3733–3742.

[B48] Zhu J.-Y. Park T. Isola P. Efros A. A. (2017). Unpaired image-to-image translation using cycle-consistent adversarial networks, in Proceedings of the IEEE International Conference on Computer Vision, Venice, 2223–2232.

